# Half-Life Extended Nanobody-Based CD38-Specific Bispecific Killercell Engagers Induce Killing of Multiple Myeloma Cells

**DOI:** 10.3389/fimmu.2022.838406

**Published:** 2022-05-16

**Authors:** Julia Hambach, William Fumey, Tobias Stähler, Anna Josephine Gebhardt, Gerhard Adam, Katja Weisel, Friedrich Koch-Nolte, Peter Bannas

**Affiliations:** ^1^ Institute of Immunology, University Medical Center Hamburg-Eppendorf, Hamburg, Germany; ^2^ Department of Diagnostic and Interventional Radiology and Nuclear Medicine, University Medical Center Hamburg-Eppendorf, Hamburg, Germany; ^3^ Department of Oncology and Hematology, University Medical Center Hamburg-Eppendorf, Hamburg, Germany

**Keywords:** bispecific engager, nanobody, multiple myeloma, BiKE, CD38, darzalex, daratumumab

## Abstract

CD38 is a target for immunotherapy of multiple myeloma. Llama-derived CD38-specific nanobodies allow easy reformatting into mono-, bi- and multispecific proteins. To evaluate the utility of nanobodies for constructing CD38-specific nanobody-based killer cell engagers (nano-BiKEs), we generated half-life extended nano-BiKEs (HLE-nano-BiKEs) by fusing a CD38-specific nanobody to a CD16-specific nanobody for binding to the Fc-receptor on NK cells and further to an albumin-specific nanobody to extend the half-life *in vivo*. HLE-nano-BiKEs targeting three different epitopes (E1, E2, E3) of CD38 were expressed in transiently transfected HEK-6E cells. We verified specific and simultaneous binding to CD38 on myeloma cells, CD16 on NK cells, and to albumin. We tested the capacity of these HLE-nano-BiKEs to mediate cytotoxicity against CD38-expressing multiple myeloma cell lines and primary myeloma cells from human bone marrow biopsies in bioluminescence and flowcytometry assays with NK92 cells as effector cells. The results revealed specific time- and dose-dependent cytolysis of CD38+ myeloma cell lines and effective depletion of CD38-expressing multiple myeloma cells from primary human bone marrow samples. Our results demonstrate the efficacy of CD38-specific HLE-nano-BiKEs *in vitro* and *ex vivo*, warranting further preclinical evaluation *in vivo* of their therapeutic potential for the treatment of multiple myeloma.

## Introduction

Multiple myeloma (MM) is a hematological disorder characterized by clonal expansion of plasma cells in the bone marrow. MM causes nearly one in eighty cancer-induced deaths worldwide (1, 2). MM leads to bone, renal, hematological, and infectious complications due to space constraints in the bone and the production of pathogenic antibodies (3). Survival of MM patients has improved with new drugs and autologous stem cell transplantation. Despite this progress, the majority of MM patients relapse (4), underlining the need for more effective treatment options with higher specificity and fewer side effects (5–7).

The NAD-metabolizing ecto-enzyme CD38 is overexpressed by MM cells and other hematological malignancies. This makes it a promising target for immunotherapies as illustrated by the approval of CD38-specific monoclonal antibodies daratumumab and isatuximab by the FDA for the treatment of relapsed MM patients (8–10).

With improvements in the field of antibody engineering, the scientific focus is shifting from conventional monoclonal CD38-specific antibodies towards recombinant antibody-based constructs, such as chimeric heavy-chain antibodies (11–14), bispecific or biparatopic constructs (15–17), chimeric antigen receptors (CARs) (18, 19), bispecific T cell engagers (BiTEs) (20, 21), and bispecific killer cell engagers (BiKEs) (22–26). BiKEs co-target the FcγRIII-Receptor CD16 on NK cells and a tumor cell surface protein, thereby engaging cytolytic NK cells to kill tumor cells (27–29).

**Figure 1:**
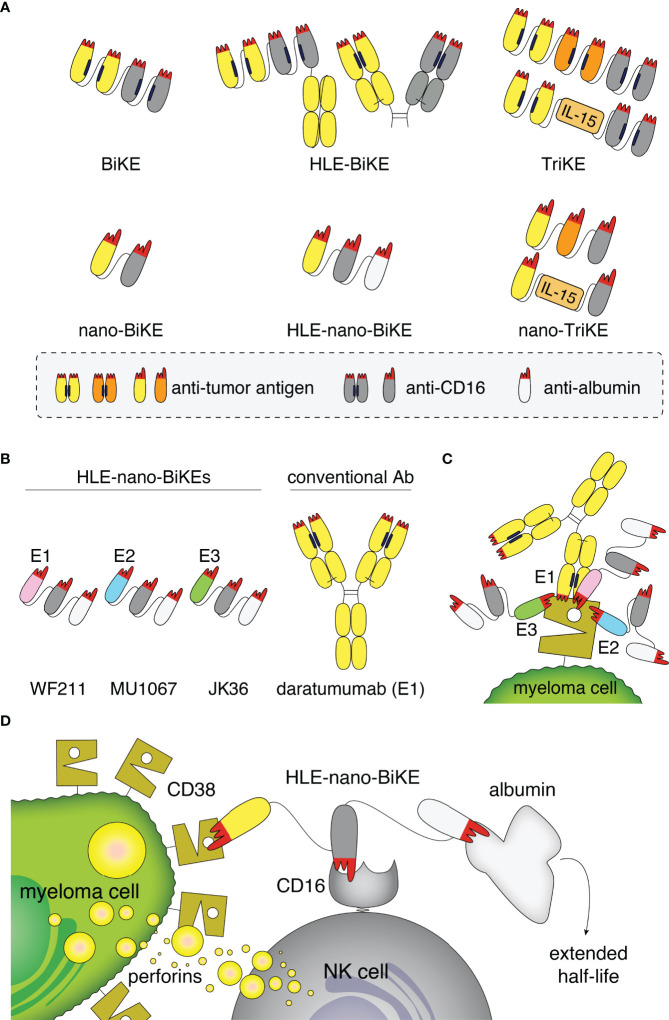
Structure, binding sites, and mode of action of half-life extended CD38-specific nanobody-based bispecific killer cell engagers (HLE-nano-BIKEs). **(A)** Scheme of bispecific and trispecific killer cell engagers (BiKEs, TriKEs) based on scFvs (top) or nanobodies (bottom). Tumor-specific and CD16-specific modules are indicated in yellow/orange and grey, respectively. Half-life extension (HLE) can be mediated by fusion to an Fc-fragment (top) or an albumin-specific nanobody (white, bottom). The paratope of each variable domain (corresponding to the CDR loops) is shown in red. Nano-TriKEs could be generated in the future by fusion of a nano-BiKE to a second tumor-specific nanobody or IL-15. **(B)** Scheme of CD38-specific HLE-nano-BiKEs (45 kDa) consisting of three nanobodies linked *via* flexible glycine-serine linkers. The N-terminal nanobody in each CD38-specific HLE-nano-BiKE recognizes one of three distinct epitopes of CD38: WF211 (pink, epitope 1 [E1]), MU1067 (blue, epitope 2 [E2]), and JK36 (green, epitope 3 [E3]). The central nanobody (grey) recognizes CD16 (FcγIII receptor on NK cells) and the C-terminal nanobody recognizes albumin (white). Conventional human antibody daratumumab (150 kDa) is indicated in yellow. **(C)** Scheme of the binding sites of daratumumab and the three HLE-nano-BiKEs. WF211-based HLE-nano-BiKE E1 recognizes an epitope (E1) that overlaps with that of daratumumab, MU1067-based HLE-nano-BiKE E2 and JK36-based HLE-nano-BiKE E3 bind independent epitopes (E2, E3). **(D)** Scheme of the proposed mode of action of a CD38-specific HLE-nano-BIKE. The N-terminal nanobody (WF211, MU1067, or JK36) binds CD38 on the myeloma cell, the central nanobody binds and activates an NK cell by targeting CD16, and the C-terminal nanobody extends the half-life of the construct by binding to albumin.

Redirecting NK cells to MM cells via a CD38-specific BiKE therefore may provide an interesting option for the treatment of MM (25, 30). BiKEs often use two single chain variable fragments (scFvs) as binding motifs. The serum half-life of scFv-based BiKEs can be extended by adding an Fc part to the scFvs or using Fab-Fragments instead of the scFvs (HLE-BiKEs). By adding a second tumor-specific scFv or IL-15 to the BiKE, a trimeric killer cell engager (TriKE) can be generated (**Figure 1A**) (31).

Nanobodies are single variable immunoglobulin domains derived from heavy chain antibodies (hcAbs) that naturally occur in camelids (32, 33). Nanobodies show high solubility and are therefore particularly suited for reformatting in a Lego brick like fashion into fusion proteins (34, 35), including heavy chain antibodies and BiKEs (36). Due to their high solubility and small size, nanobody-based BiKEs (nano-BiKEs, ca. 30kDa) might provide better stability and higher tissue penetration in vivo than scFv-based BiKEs (ca. 50 kDa) (**Figure 1A**) (33, 37). However, the small size of nanobody dimers lies below that of the renal filtration barrier, accounting for a short serum half-life in vivo. This can be overcome by fusion of a nanobody-based BiKE to an albumin-specific nanobody (38–40), allowing nanobody-trimers, e.g. half-life extended nano-BiKEs (HLE-nano-BiKEs) (**Figure 1A**), to “piggy-back” on circulating albumin, thereby hindering renal filtration (41).

The aim of this study was to explore the utility of nanobodies recognizing three non-overlapping epitopes on CD38 for constructing CD38-specific HLE-nano-BiKEs and to evaluate their therapeutic potential *in vitro* and *ex vivo*.

## Materials and Methods

### Cell Lines

Human cell lines NK92 (natural killer cell line), OPM-2 and LP-1 (myeloma cell lines), and CA-46 and Daudi (Burkitt lymphoma cell lines) were obtained from the German Collection of Microorganisms and Cell Culture (DSMZ, Braunschweig, Germany). We generated OPM-2 luc, LP-1 luc, CA-46 luc, and Daudi luc cell lines stably expressing the luc2 variant of *Photinus pyralis* luciferase (Promega, Madison, WI) under control of the spleen-focus-forming virus U3 region (SFFV promoter) by lentiviral transduction (15). HEK-6E cells (42) were kindly provided by Yves Durocher, Ottawa, Canada.

Transduced cells were selected in culture medium containing 1 µg/mL puromycin and subsequently sorted by FACS based on eGFP expression (FACS Aria III, BD Biosciences, Heidelberg, Germany). Sorted cells were kept in culture and luciferase-expression was controlled regularly following addition of luciferin using a luminometric plate reader. NK92 cells were stably transfected with human CD16 and eGFP by retroviral transduction using the pSF91 vector. The sequence for CD16, i.e. the ectodomain of Fc*γ*RIII fused to the transmembrane and cytosolic domains of FcϵRI was kindly provided by Béatrice Clémenceau, Nantes, France (43). The CD38 gene in NK92, NK92 hCD16, and LP-1 luc cells was inactivated using CRISPR/Cas9 technology (sc-401117-NIC, Santa Cruz Biotechnology) as previously described (15). Cell surface levels of CD38 were determined by staining for 30 min at 4°C with CD38-specific AlexaFluor647-conjugated JK36 hcAb or the ARTC2.2-specific s-14 hcAb as isotype control.

### Generation of Nanobody-Based BiKEs

The human CD38-specific nanobodies WF211, MU1067, JK36 and the ARTC2.2-specific control nanobody s-14 (co) were generated from immunized llamas as described previously (44–46). WF211 binds Epitope 1 (E1), MU1067 binds Epitope 2 (E2), and JK36 binds Epitope 3 (E3) on CD38. For the sake of clarity, we use abbreviated designations for HLE-nano-BiKEs in the figures, e.g., HLE-nBiKE E1, HLE-nBiKE E2, and HLE-nBiKE E3 (**Figures 1B, C**).

The sequence coding for the CD16-specific nanobody c21 was obtained from published work by Ghislaine Behar, Paris, France (47). The sequence of the albumin-specific nanobody Alb11 was obtained from patent US20070269422A1. Nanobody Alb11 mediates *in vivo* half-life extension of monomeric and dimeric nanobodies by retarding renal filtration (48). The respective off-rates (k_diss_) of these nanobodies have been published: WF211 4.5x10^-3^ (s^-1^), JK36 2x10^-4^ (s^-1^), MU1067 1.2x10^-4^ (s^-1^) (44), c21 2.3x10^-3^ (s^-1^) and Alb11 9.8x10^-4^ (s^-1^) (47, 48).

HLE-nano-BiKE E1, HLE-nano-BiKE E2, HLE-nano-BiKE E3, and isotype control HLE-nano-BiKE (HLE-nano-BiKE co) were generated by subcloning the coding region for the respective CD38-specific nanobody or control nanobody upstream of the coding regions for c21 and Alb11, with intervening, flexible gly-ser linkers and cloned into the pCSE2.5 vector [kindly provided by Tim Schirrmann, Braunschweig, Germany (49)]. HLE-nano-BiKEs were expressed in transiently transfected HEK-6E cells (50) cultivated in serum-free medium as described previously (51). Supernatants were harvested six days post transfection. HLE-nano-BiKEs were purified from the supernatants by affinity chromatography using protein A sepharose (51). Purity of antibody constructs was assessed by SDS-PAGE and InstantBlue™ Coomassie staining. Daratumumab (Darzalex) was purchased from Janssen-Cilag, Neuss, Germany, to be used as positive control in our killing assays. Biotinylated human albumin (ab8033) was purchased from Abcam, Cambridge, United Kingdom.

### Binding of BiKEs

Binding of HLE-nano-BiKEs was assessed by incubation of LP-1 luc cells or NK92 hCD16 CD38KO cells with 100 nM HLE-nano-BiKEs for 15 minutes. To detect specific binding of HLE-nano-BiKEs, biotinylated human albumin was added and detected with PE-Cy7 conjugated streptavidin (Becton Dickinson, NJ, USA). Control staining was performed with albumin and PE-Cy7-conjugated streptavidin alone. Cell-associated fluorescence was determined by flow cytometry.

### Biolayer Interferometry

The extracellular domain of human CD38 (aa 46–300) was produced as a secretory protein with a His6x-Myc epitope tag in transiently transfected HEK-6E cells. The tagged protein was purified using immobilized metal affinity chromatography (IMAC). The purified protein was biotinylated using the EZ-Link™ Sulfo-NHS-LC-Biotin (A39257) from Thermo Fisher Scientific (Waltham, MA, USA) according to manufacturer’s instructions. Recombinant CD16a (A42536) was purchased from Thermo Fisher Scientific (Waltham, MA, USA). Human albumin (Alburex^®^ 20) was purchased from CSL Behring (PA, USA).

Binding of the HLE-nano-BiKEs to CD38, CD16a, and albumin was determined by BLI-technology using a fortéBIO BLItz instrument. Assays were performed at 20°C with running buffer (PBS, 0.002% (v/v) Tween-20). Streptavidin sensors were hydrated in running buffer and loaded for 30 seconds with biotinylated CD38 (3.7 µM). After washing for 60 sec purified HLE-nano-BiKEs (E1, E2, E3, or control) (1.33 µM) were allowed to associate for 60 sec on immobilized CD38, followed by dissociation for 45 sec. Then, binding of CD16 (0.8 µM) to the bound BiKE was allowed for 60 sec followed by a 45 sec dissociation phase. Finally, albumin (6 µM) was added for 60 sec followed by 120 sec of dissociation. Curve fitting and affinity calculations were performed using Graph Pad Prism (version 7).

### BiKE-Dependent Cellular Cytotoxicity (BiKE-DCC)

Cytotoxicity of CD38-specific HLE-nano-BiKEs was assessed using OPM-2 luc, LP-1 luc, CA-46 luc, or Daudi luc cells expressing CD38 and LP-1 luc CD38KO cells as negative control. NK92 hCD16 cells were used as effectors with NK92 cells as control for specificity of NK92 hCD16 cell activation through CD16. To distinguish cells in mixed populations, cells were strategically labeled with eFluor450 or eFluor670. LP-1 luc CD38KO cells were labeled with eFluor670, washed and then mixed with unlabeled LP-1 luc cells in a 1:1 ratio. Next, the indicated concentrations of HLE-nano-BiKEs were added and 15 min later, NK92 or NK92 hCD16 cells were added in the indicated effector to target ratio. Cells were incubated in αMEM culture medium supplemented with 10% fetal calf serum (FCS), 10% horse serum, 5 mM glutamine, and 5 ng/ml interleukin 2 (IL-2 Proleukin-S, Novartis, Basel, Switzerland) at 37°C for 3 h. Assays were performed without and in the presence of 16 mg/mL human albumin as indicated. Cells were then stained with 20 µg/mL propidium iodide (PI) to identify dead cells and analyzed by flow cytometry. Alternatively, D-luciferin (Biosynth, Staad, Switzerland) was added as substrate (75 µg/ml) for 20 min and bioluminescence-intensity (BLI) was measured using a microplate reader (Victor³, Perkin Elmer, Boston, USA). Percentage of lysed cells was calculated as follows:


$$percent\;of\;BLI\;\left[\%\right]=\left(\frac{BLI\left[sample\right]}{BLI\left[sample\;w/o\;BiKE\right]}\right)x100\%$$

For kinetic analyses, NK92 hCD16 cells were pre-incubated for 30 min with 100 nM HLE-nano-BiKEs or daratumumab and unbound constructs were removed by centrifugation. LP-1 luc were added at different ratios in αMEM culture medium supplemented with 10% FCS, 10% horse serum, 5 mM glutamine, and 5 ng/ml interleukin 2. D-luciferin (Biosynth, Staad, Switzerland) was added (75 µg/ml). Cells were incubated at 37°C and measurement of BLI was performed using a microplate reader (Victor³, Perkin Elmer, Boston, USA). Percentage of lysed cells was calculated as described above.

Fresh bone marrow aspirates were obtained from patients after Institutional Review-Board-approved consent (PV5505). Bone marrow mononuclear cells (BM-MNCs) were prepared by Ficoll-Paque density gradient centrifugation and subsequent depletion of remaining erythrocytes using red blood cell lysis buffer (NH4Cl + KHCO3 + EDTA). BM-MNCs were pre-incubated with 10 nM HLE-nano-BiKEs or daratumumab for 15 min before addition of NK92 hCD16 cells in an effector to target ratio of 3:1. Cells were then stained with a panel of fluorochrome-conjugated antibodies (CD38, CD45, CD138/229, CD269/CD319/CD56, CD19) and the viability dye Pacific Orange and analyzed *via* flow cytometry. Staining of CD38 was achieved with AlexaFluor647-conjugated nanobodies that bind independently of the nanobody contained in the HLE-nano-BiKE, i.e. JK36^AF647^ or MU523^AF647^ for HLE-nano-BiKE E1 and daratumumab, MU523^AF647^ or WF211^AF647^ for HLE-nano-BiKE E2, and JK36^AF647^ or WF211^AF647^ for HLE-nano-BiKE E3. An FSC threshold was set to exclude debris while including the population of small CD19^+^ B cells. Dead cells were excluded using PacO staining. NK92 hCD16 cells were excluded by eGFP-expression. MM cells were identified by co-expression of CD138, and CD38. Numbers of MM cells were determined using CountBright absolute counting beads (Invitrogen). Percentage of lysed MM cells was calculated as follows:


$$percent\;of\;lysis\;\left[\%\right]=\left(1-\left(\frac{MM\;cell\;number\;per\;\text{µ}L\left[sample\right]}{MM\;cell\;number\;per\;\text{µ}L\left[sample\;w/o\;BiKE\right]}\right)\right)x100\%$$

Significant differences in surviving cells treated with CD38-specific HLE-nano-BiKEs vs. control HLE-nano-BiKE was calculated using One-way ANOVA followed by a Holm-Sidak test (GraphPad Prism, GraphPad Software, CA, USA).

## Results

### Specific and Simultaneous Binding of HLE-nano-BiKEs to CD38, CD16,and Albumin

**Figure 2:**
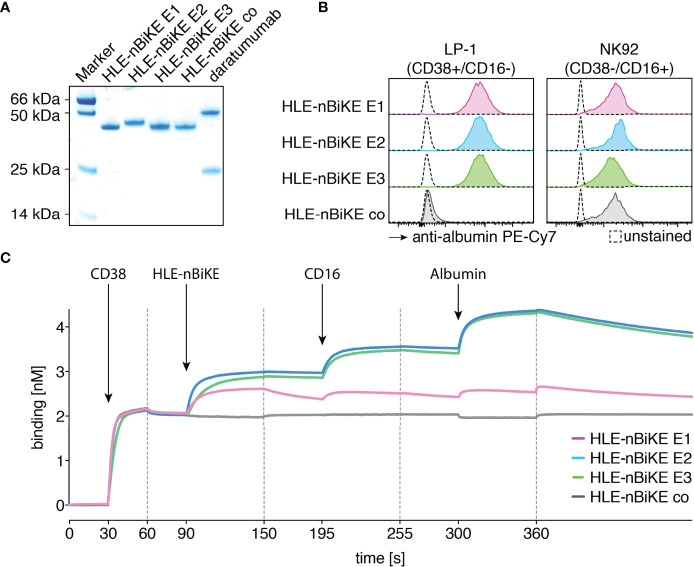
Purified CD38-specific HLE-nano-BiKEs bind specifically and simultaneously to myeloma cells, NK cells, and albumin. **(A)** Purified HLE-nano-BiKEs and daratumumab (1 µg per lane) were size fractionated by SDS-PAGE under reducing conditions and visualized by Coomassie staining. **(B)** LP-1 luc myeloma cells (left panel) and NK92 hCD16 cells (right panel) were incubated with CD38-specific HLE-nano-BiKE E1, HLE-nano-BiKE E2, HLE-nano-BiKE E3, or an isotype control (HLE-nano-BiKE co). Bound HLE-nano-BiKEs were detected by flow cytometry using biotinylated human albumin and PE-Cy7-conjugated streptavidin by flow cytometry. Control stainings (unstained, dashed line) were performed with albumin and PE-Cy7-conjugated streptavidin alone. Results are representative of three similar experiments. **(C)** BLI analyses of the sequential binding of HLE-nano-BiKEs, CD16 and albumin to immobilized CD38. The biotinylated recombinant ectodomain of human CD38 was bound to a streptavidin-coated BLI sensor (30-90s). HLE-nano-BiKEs, recombinant ectodomain of human CD16, and human albumin were added at the times indicated. In each case binding was measured for 60s with a subsequent 60s dissociation phase. Arrows indicate the time point of protein addition; dashed lines indicate the start of the dissociation phase.

We purified HLE-nano-BiKEs from the supernatants of transiently transfected HEK-6E cells using protein A and verified the integrity and purity of these constructs by SDS-PAGE and Coomassie staining (**Figure 2A**). Specific binding of HLE-nano-BiKEs to CD38 and CD16 was analyzed on CD38+/CD16- LP-1 cells and CD38-/CD16+ NK92 cells. Bound HLE-nano-BiKEs were detected with biotinylated albumin and PE-conjugated Streptavidin. This staining strategy allowed us to verify the functionality of the albumin-specific nanobody used in the trimeric construct (**Figure 2B**). Regardless of their epitope specificity (E1, E2, or E3), all three CD38-specific HLE-nano-BiKEs (E1, E2, and E3) bound CD38-expressing LP-1 luc cells. The control HLE-nano-BiKE co did not bind to CD38-expressing LP-1 luc cells, substantiating the specific binding of the CD38-specific HLE-nano-BiKEs *via* their CD38-specific nanobodies (WF211, MU1067, or JK36) to CD38 on the surface of LP-1 cells.

The three CD38-specific HLE-nano-BiKEs and the isotype-control HLE-nano-BiKE bound to NK92 hCD16 CD38KO cells. This verifies specific binding of the CD16-specific nanobody in the trimeric BiKE to CD16 on the effector NK92 cells. Further, the detection system with biotinylated human albumin confirmed the functionality of the albumin-specific nanobody in our HLE-nano-BiKEs.

Biolayer interferometry was used to determine whether HLE-nano-BiKEs allow for simultaneous binding of CD38, CD16, and albumin (**Figure 1D**). After binding of biotinylated CD38 to a streptavidin sensor, HLE-nano-BiKEs (E1, E2, or E3), recombinant CD16, and albumin were added sequentially. The results show successive incremental increases in signal intensities upon addition of a CD38-specific HLE-nano-BiKE, CD16, and allow for simultaneous binding of CD38, CD16 and albumin (**Figure 2C**). HLE-nano-BiKEs E2 and E3 showed comparable and relatively high signal intensities. Signal increase was lower for HLE-nano-BiKE E1. HLE-nano-BiKE E1 contains the CD38-specific nanobody WF211, which has a lower affinity for CD38 than nanobodies MU1067 (HLE-nano-BiKE E2) and JK36 (HLE-nano-BiKE E3) (44). The isotype-control HLE-nano-BiKE showed no binding to CD38. These results demonstrate that CD38-specific HLE-nano-BiKEs can simultaneously bind CD38, CD16, and albumin.

### CD38-Specific HLE-nano-BiKEs Specifically Induce NK92 Cell-Mediated Cytolysis of the CD38^+^ LP-1 Myeloma Cell Line

**Figure 3:**
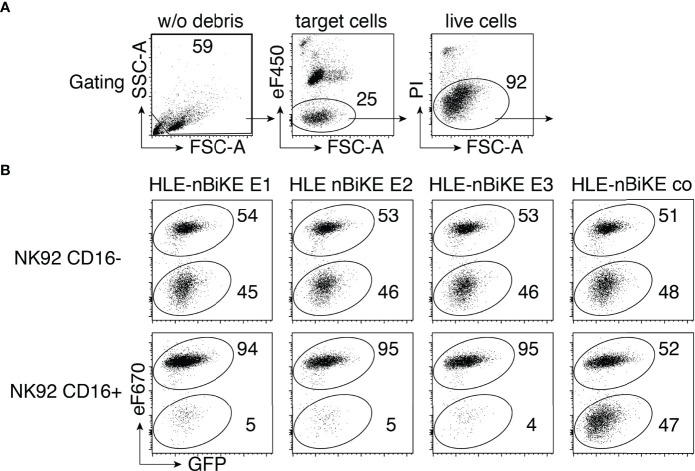
CD38-specific HLE-nano-BiKEs specifically induce NK cell-mediated cytotoxicity toward CD38+ myeloma cells. **(A)** Effector NK92 cells were pre-labeled with eFluor450 (eF450). Gating was performed to exclude cellular debris (lower left corner) of panel 1, and then on target cells, identified as eF450-negative cells (panel 2). Dead cells were excluded by PI-staining. **(B)** CD38KO GFP+ LP-1 myeloma cells labeled with eFluor670 (eF670) (upper population) were mixed with unlabeled CD38-expressing GFP+ LP-1 cells (lower population) and the indicated HLE-nano-BiKEs before addition of eF450-labeled NK92 cells stably transfected with human CD16 (lower panels) or lacking CD16 (upper panels, negative control) at an effector-to-target ratio of 3:1. Cells were incubated for 3 h at 37°C and analyzed by flow cytometry in the presence of the DNA staining dye propidium iodide (PI). Loss of cells from the alive cells was used to monitor HLE-nano-BiKE-induced lysis of target cells. Gating was performed on target cells as described in **(A)** Numbers indicate percentage of cells in each quadrant. Results are representative of three similar experiments.

The cytotoxic effect of CD38-specific HLE-nano-BiKEs was tested on LP-1 luc myeloma cells (**Figure 3**). We performed BiKE-dependent cellular cytotoxicity (BiKE-DCC) assays on a mixed suspension of GFP^+^/CD38^+^ and GFP^+^/CD38KO LP-1 luc cells to control for specificity of BiKE-DCC to CD38 expressing target cells. Cytolysis was assessed by uptake and staining of cells for the DNA-binding dye propidium iodide. In parallel, we performed a BiKE-DCC assay with NK92 cells that lack cell surface CD16 to assess the dependency of BiKE-DCC on CD16.

The results reveal that all three CD38-specific HLE-nano-BiKEs specifically induce the killing of CD38-expressing LP-1 myeloma cells, but not of CD38KO LP-1 myeloma cells. Neither the isotype-control HLE-nano-BiKE co nor CD16-negative NK92 cells mediated killing of LP-1 myeloma cells. These results strongly suggest that the specific cytotoxic effect of our HLE-nano-BiKE is mediated by cross-linking CD38 on target myeloma cells with CD16 on NK92 effector cells.

### HLE-nano-BiKEs Induce Cytolysis of Myeloma Cells by NK92 Cells in a Dose and Effector to Target-Ratio Dependent Manner

**Figure 4:**
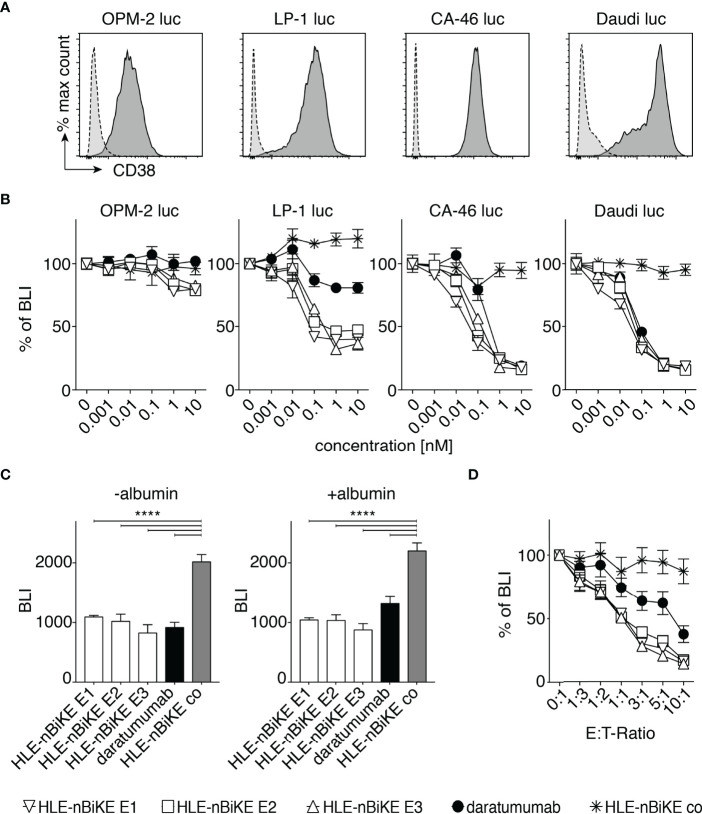
Dose and effector to target-ratio (E:T-ratio) responses of BiKE-DCC. **(A)** Luciferase (luc) expressing OPM-2, LP-1, CA-46, and Daudi cells were incubated with AlexaFluor 647-conjugated CD38-specific JK36 hcAb (dark grey, solid line) or isotype control s-14 hcAb (light grey, dashed line) before analysis by flow cytometry. **(B)** NK92 hCD16 cells were incubated an E:T-ratio of 3:1 with OPM-2 luc, LP-1 luc, CA-46, or Daudi luc cells that had been pre-incubated with the indicated concentrations of HLE-nano-BiKEs or daratumumab for 3 h at 37°C before addition of luciferin and measurement of bioluminescence-intensity (BLI) with a plate reader. Data represent mean ± SEM of triplets and are representative for three independent experiments. **(C)** LP-1 luc cells were incubated for 15 min with 10 nM HLE-nano-BiKEs or daratumumab in the absence or presence of 16 mg/mL albumin. Then, NK92 hCD16 cells were added at an E:T-ratio of 3:1 and incubation continued for 95 min at 37°C before addition of luciferin and measurement of BLI. Bar diagrams illustrate the mean BLI of cells after incubation with HLE-nano-BiKEs or daratumumab. One-way ANOVA was performed and Holm-Sidak-adjusted p-values are indicated (****= p < 0.0001). **(D)** NK92 hCD16 cells were incubated at the indicated E:T-ratios (0:1-1:10) with LP-1 luc cells that had been pre-incubated with 10 nM HLE-nano-BiKEs or daratumumab and BLI was measured after 3 h of incubation at 37°C. Values indicate the mean BLI of cells incubated with 10 nM HLE-nano-BiKEs or daratumumab as percentage of the mean BLI of cells incubated in the absence of HLE-nano-BiKEs.

We next set out to assess the efficacy of BiKE-DCC and antibody-dependent cellular cytotoxicity (ADCC) mediated by daratumumab. For this we assessed the dependency of cytolysis on the concentration of HLE-nano-BiKEs or daratumumab and on the ratio of hCD16 effector cells to myeloma target cells (E:T-ratio). We used a luminescence-based assay with luciferase-transduced OPM-2 luc, LP-1 luc, CA-46 luc, and Daudi luc cells as target cells (**Figure 4**). These cell lines show moderate to high levels of CD38 on the cell surface (**Figure 4A**).

For assessment of BiKE-DCC, OPM-2 luc, LP-1 luc, CA-46 luc, and Daudi luc cells were incubated with increasing concentrations (0 nM to 10 nM) of HLE-nano-BiKEs or daratumumab and NK92 hCD16 cells at an E:T-ratio of 3:1. After 3h, luciferin was added and the BLI-Signal was measured (**Figure 4B**). The results show that the three CD38-specific HLE-nano-BiKEs mediate NK-cell cytotoxicity against all cell lines in a dose dependent fashion. BiKE-DCC and daratumumab induced ADCC was lowest in OPM-2 luc cells, consistent with the relatively low cell surface levels of CD38 on these cells (**Figure 4A**).

Next, we aimed to determine whether the presence of albumin could impair HLE-nano-BiKE induced killing, i.e. by binding to the albumin-binding nanobody Alb11. LP-1 luc cells were incubated for 90 min with 10 nM HLE-nano-BiKEs or daratumumab and NK92 hCD16 cells at an E:T-ratio of 3:1 in the absence or presence of 16mg/mL albumin. The results show that all three CD38-specific HLE-nano-BiKEs (E1, E2, E3) as well as daratumumab effectively mediated NK-cell cytotoxicity against LP-1 myeloma cells. The presence of albumin impaired neither BiKE-DCC nor daratumumab mediated ADCC (**Figure 4C**).

We next compared the efficacies of BiKE-DCC vs. ADCC at different E:T-ratios using a saturating dose of 10nM BiKEs or daratumumab. The results again indicate that BiKE-DCC is more effective than ADCC (**Figure 4D**). The same degree of cytolysis was achieved at 3-10 fold lower E:T-ratios with CD38-specific HLE-nano-BiKEs than with daratumumab. At this dose (10 nM) the isotype-control HLE-nano-BiKE co did not induce killing of myeloma cells, even at the highest E:T-ratio of 10:1.

### Kinetics of CD38-Specific HLE-nano-BiKE-Induced Killing of Myeloma Cells

**Figure 5:**
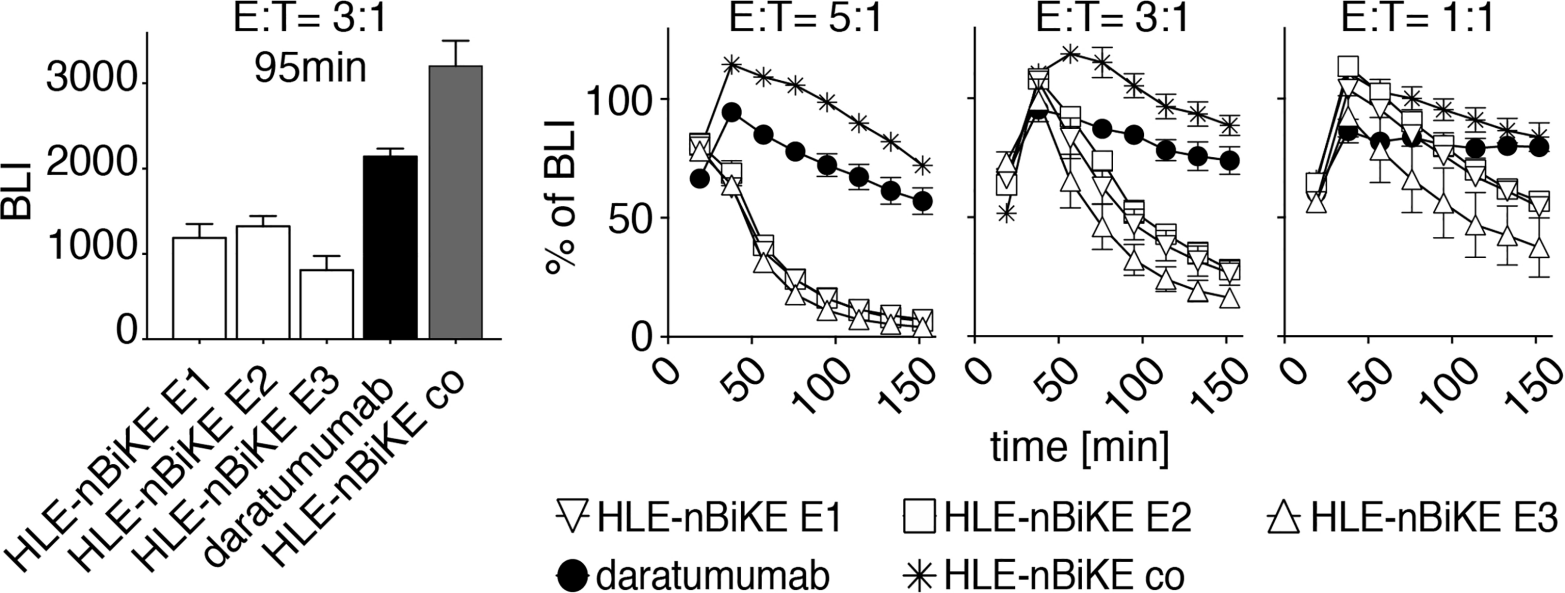
Kinetics of CD38-specific HLE-nano-BiKE-induced killing of myeloma cells. CD16^+^ NK92 hCD16 cells were incubated with nanobody-based CD38-specific HLE-nano-BiKEs (E1, E2, E3), isotype-control HLE-nano-BiKE co, or daratumumab and then added to luciferase-expressing LP-1 luc myeloma cells at the indicated effector to target ratios. Mean absolute BLI signal is shown in the left panel at an E:T-ratio of 3:1 after 95 min of incubation. At timepoint t= 0min NK cells were added to LP-1 cells preincubated with luciferin. Kinetics (0 - 150 min) of HLE-nano-BiKE-induced killing is shown as percentage of BLI in comparison to myeloma cells treated without HLE-nano-BiKEs in the right panel. Data represent mean ± SEM of triplets. Results are representative of three similar experiments.

Next, we analyzed the kinetics of cytolysis induced by HLE-nano-BiKEs vs. ADCC induced by daratumumab using the saturating dose of 10 nM and E:T ratios of 5:1, 3:1, and 1:1 (**Figure 5**). The results reveal a much faster cytolysis induced by HLE-nano-BiKEs than by daratumumab. Again, the three CD38-specific HLE-nano-BiKEs recognizing different epitopes (E1, E2, E3) of CD38 induced cytolysis with a similar, time-dependent efficacy (**Figure 5**). Daratumumab also induced cytolysis in a time-dependent manner, but much delayed compared to the three HLE-nano-BiKEs, and again without reaching a maximal degree of cytolysis. Cells incubated with isotype-control HLE-nano-BiKE co and NK92 hCD16 cells showed a low degree of time-dependent cytolysis, especially at later time points, likely reflecting background (i.e. unspecific) cell death.

### CD38-Specific HLE-nano-BiKEs Induce NK92 Cell Mediated Cytolysis of Primary Myeloma Cells

**Figure 6:**
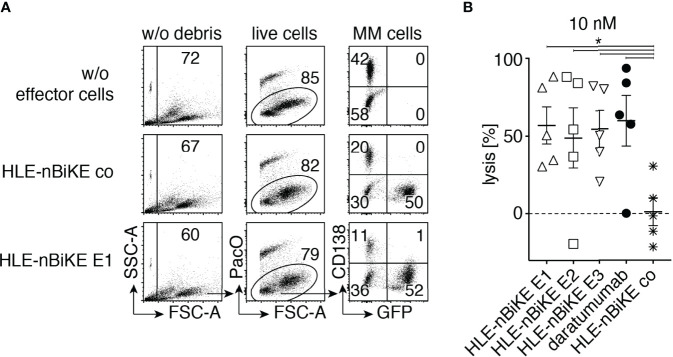
CD38-specific HLE-nano-BiKEs deplete CD38+ primary myeloma cells from MM patients. Fresh primary human bone marrow mononuclear cells were incubated for 15 min in the absence or presence of 10 nM HLE-nano-BiKEs or daratumumab before addition of GFP-transfected NK92 hCD16 effector cells at an effector to target ratio of 3:1 and further incubation for 3 h at 37°C. Counting beads were added and cells were counterstained with Pacific Orange (PacO) and brilliant violet 421-conjugated CD138-specific mAb before analysis by flow cytometry. **(A)** Gating was performed on live cells by excluding beads (SSC-hi/FSC-lo) and debris (SSC-lo/FSC-lo) (left panels), doublets (not shown), and dead cells (middle panels). Myeloma cells and NK92 hCD16 effector cells were identified by their expression of CD138 and GFP, respectively. Numbers indicate the percentage of cells in the gated population. **(B)** The absolute number of surviving myeloma cells (GFP-/CD138+/CD38+) was determined with the aid of cell counting beads. Percentages of surviving myeloma cells were calculated relative to the number of surviving myeloma cells in samples incubated without HLE-nano-BiKEs (set at 100%) to calculate lysis. Data represent means ± SEM. One-Way ANOVA was performed and Holm-Sidak-adjusted p-values are indicated (*= p < 0.05).

In a final set of experiments, we assessed the efficacy of BiKE-DCC compared to ADCC mediated by daratumumab against primary multiple myeloma cells from bone marrow samples of five myeloma patients, at 10 nM of HLE-nano-BiKEs or daratumumab and an E:T ratio of ~ 3:1 (**Figure 6**). MM cells were identified based on high cell surface levels of CD38 and CD138. The infiltration of MM cells varied from 3 to 35% (mean 13%). We excluded debris and dead cells based on low forward scatter and Pacific Orange-staining and NK92 hCD16 cells by their GFP-expression. Counting beads were added to the samples to permit quantification of absolute cell numbers. The results show that the CD38-specific HLE-nano-BiKEs and daratumumab induced NK92 hCD16 cell mediated cytolysis of primary CD138+/CD38+ MM cells with similar efficacies, while the isotype-control HLE-nano-BiKE co did not induce NK92 hCD16 cell mediated death of primary myeloma cells.

## Discussion

Our results demonstrate the feasibility of using CD38-specific HLE-nano-BiKEs to efficiently kill MM and Burkitt lymphoma cells. CD38-specific HLE-nano-BiKEs that recognize three different and non-overlapping epitopes of CD38 all induced potent cytotoxicity of tumor cell lines *in vitro* and of primary MM cells *ex vivo*.

Our CD38-specific HLE-nano-BiKEs all showed specific binding to CD38 on myeloma cells, CD16 on NK cells, and to human albumin. BiKE-DCC by HLE-nano-BiKEs was shown to rely on binding to both, CD38 on target myeloma cells and CD16 on effector NK cells. BiKE-DCC was dose- and time-dependent and effected by the E:T-ratio of NK effector cells to myeloma target cells. NK92 hCD16-mediated cytolysis was induced more effectively by our nanobody-based BiKEs (BiKE-DCC) than by the conventional CD38-specific antibody daratumumab (ADCC). Our results are in line with a recent publication by van Faassen et al., describing BiKEs directed against CD19, HER2 or EGFR that induced effective BiKE-DCC against cells that display the respective target on the cell surface (36).

Our trimeric HLE-nano-BiKEs were readily produced, purified and concentrated without showing any signs of aggregation. These features might overcome problems encountered previously with bispecific engagers constructed of scFvs, which are often difficult to produce, exhibit low solubility, and/or a tendency to aggregate, leading to developability issues (52–55). Key advantages of HLE-nano-BiKEs over scFv-based BiKEs are their high solubility, easy reformability, and small size. Moreover, our HLE-nano-BiKEs harness the advantages of the nanobody format for half life extension by linkage of the BiKE to an additional albumin-binding nanobody, rather than to a larger Fc-fragment (**Figure 1A**).

The albumin-specific nanobody in our HLE-nano-BiKE-constructs is meant to extend the half-life of the construct *in vivo* as shown in previous studies (38–40). Though the increased half-life of our CD38-specific HLE-nano-BiKEs *in vivo* remains to be shown, we could show that the albumin-specific nanobody used in our constructs binds to human albumin *in vitro* and that binding to albumin does not impair the capacity to mediate BiKE-DCC.

The three epitopes (E1, E2, E3) of CD38 on MM cells addressed by our HLE-nano-BiKEs have important clinical implications. Two of these HLE-nano-BiKEs (E2, E3) bind to epitopes on CD38 that are independent (E2, E3) of that of daratumumab (E1) (44). These two HLE-nano-BiKEs could therefore be used in MM patients that have been treated with daratumumab without being blocked from binding to CD38 by daratumumab (56, 57). Similarly, two of these HLE-nano-BiKEs (E1, E2) bind to epitopes on CD38 that are independent (E1, E2) of that of isatuximab (E3) and could therefore be used potentially in MM patients that have been treated with isatuximab.

Our HLE-nano-BiKEs are composed only of lama-derived variable immunoglobulin domains (VHH or nanobody). Similar to therapeutics that incorporate murine VH and VL domains such as blinatumumab, a CD19-CD3 bispecific T-cell engager composed of two murine scFv (58, 59) or rituximab (60), a chimeric antibody composed of murine VH and VL and human constant IgG1 domains, therapeutics composed of VHH domains such as caplacizumab (61) (a dimer of two llama VHH domains) show little if any immunogenicity in patients (62). Anti-drug antibodies in patients can abrogate the efficacy of antibody treatment; therefore antibody-constructs with a low immunogenicity are favorable (63–65). Moreover, the better solubility and the smaller size of an HLE-nano-BiKE (ca. 45kDa) in comparison to an scFv-based BiKE (ca. 55kDa) could additionally provide better tissue penetration *in vivo* (36, 37).

The major limitation of our proof-of-concept *in vitro* and *ex vivo* study is the lack of the assessment of cytotoxic effects of our CD38-specific HLE-nano-BiKEs in *in vivo* xenograft myeloma mouse models. These *in vivo* studies are warranted as follow-up experiments after further optimization of our HLE-nano-BiKEs *in vitro*. We aim to examine the effect of the length of the two linkers and the order of the three nanobodies on the effectiveness of the BiKE-DCC. Van Faassen et al. observed only small effects by changing the order of the nanobodies and the lengths of the linker in their nano-BiKEs (36). Notwithstanding, we hypothesize that the length of the linker may differentially affect the efficacy of HLE-nano-BiKEs that target different epitopes of CD38.

In summary, we here provide proof of principle for the efficacy of CD38-specific HLE-nano-BiKEs *in vitro* and *ex vivo*, warranting further preclinical evaluation *in vivo* of their therapeutic potential for the treatment of multiple myeloma.

## Data Availability Statement

The raw data supporting the conclusions of this article will be made available by the authors, without undue reservation.

## Ethics Statement

The studies involving human participants were reviewed and approved by Hamburger Ärztekammer. The patients/participants provided their written informed consent to participate in this study.

## Author Contributions

PB and FK-N conceived the project. All authors established experimental procedures. JH, PB, and FK-N wrote the manuscript. All authors contributed to the article and approved the submitted version.

## Funding

Supported by grants from the Deutsche Forschungsgemeinschaft to PB (BA 5893/7) and FK-N (No310/16 and SFB1328- Z02).

## Conflict of Interest

FK-N receives a share of antibody sales *via* MediGate GmbH, a wholly owned subsidiary of the University Medical Center Hamburg-Eppendorf. WF, FK-N, and PB are co-inventor on a patent application on CD38-specific nanobodies.

The remaining authors declare that the research was conducted in the absence of any commercial or financial relationships that could be construed as a potential conflict of interest.

## Publisher’s Note

All claims expressed in this article are solely those of the authors and do not necessarily represent those of their affiliated organizations, or those of the publisher, the editors and the reviewers. Any product that may be evaluated in this article, or claim that may be made by its manufacturer, is not guaranteed or endorsed by the publisher.
